# Co-design of a decision aid for the management of lentigo maligna in older and frailer adults

**DOI:** 10.1093/skinhd/vzaf118

**Published:** 2026-03-20

**Authors:** Dimitrios Karponis, Elisa Cardamone, Khaylen Mistry, Frank Halliday, Martyn Patel, Nick J Levell, Alexander J Stratigos, Vasiliki Nikolaou, Zoe C Venables

**Affiliations:** Norwich Medical School, University of East Anglia, Norwich, UK; Department of Dermatology, Norfolk and Norwich University Hospital, Norwich, UK; 1st Department of Dermatology-Venereology, National and Kapodistrian University of Athens, Andreas Sygros Hospital, Athens, Greece; Advanced Care Research Centre, Usher Institute, University of Edinburgh, Edinburgh, UK; Norwich Medical School, University of East Anglia, Norwich, UK; Department of Dermatology, Norfolk and Norwich University Hospital, Norwich, UK; Department of Dermatology, Norfolk and Norwich University Hospital, Norwich, UK; Norwich Medical School, University of East Anglia, Norwich, UK; Department of Older People’s Medicine, Norfolk and Norwich University Hospital, Norwich, UK; Norwich Medical School, University of East Anglia, Norwich, UK; Department of Dermatology, Norfolk and Norwich University Hospital, Norwich, UK; 1st Department of Dermatology-Venereology, National and Kapodistrian University of Athens, Andreas Sygros Hospital, Athens, Greece; 1st Department of Dermatology-Venereology, National and Kapodistrian University of Athens, Andreas Sygros Hospital, Athens, Greece; Norwich Medical School, University of East Anglia, Norwich, UK; Department of Dermatology, Norfolk and Norwich University Hospital, Norwich, UK; National Disease Registration Service, Data and Analytics, NHS Digital, Leeds, UK

## Abstract

**Background:**

Lentigo maligna (LM) can be treated surgically, topically using imiquimod, or simply observed. The risk of progression to melanoma is considered low. There is clinical equipoise on management in older or frail adults, with no decision aids available for managing LM in this group.

**Objectives:**

To co-design a patient decision aid (PDA) for the management of LM in older or frail adults.

**Methods:**

The PDA was targeted at patients at least 85 years old, or those aged 75–84 years with a Rockwood Clinical Frailty Score of at least 4. Semi-structured interviews were conducted with patients and healthcare practitioners (HCPs) to co-design the tool. Thematic analysis was performed using NVivo 14. The prototype was tested via structured interviews with patients and HCPs, and amended in an iterative process until saturation was achieved.

**Results:**

In total, 13 patients and 18 HCPs were interviewed to co-design the prototype. Alpha-testing resulted in saturation after 20 iterations. The following key themes (and subthemes) were identified Theme 1: fear of cancer (definition of LM; risk of progression; prognosis); Theme 2: burden of treatment (risks, side effects and delays in treatment; likelihood of recurrence; frequency of follow-up; impact on quality of life); Theme 3: partnership and shared decision-making (empowering patients; involving family and carers; burden of responsibility); and Theme 4: information overload (less is more; accessibility).

**Conclusions:**

This is the first PDA for the management of LM in older and frail patients. This co-designed, patient-centred tool may empower patients to participate in decision-making by better understanding LM and the available management options.

## Graphical Abstract

**Graphical Abstract:**
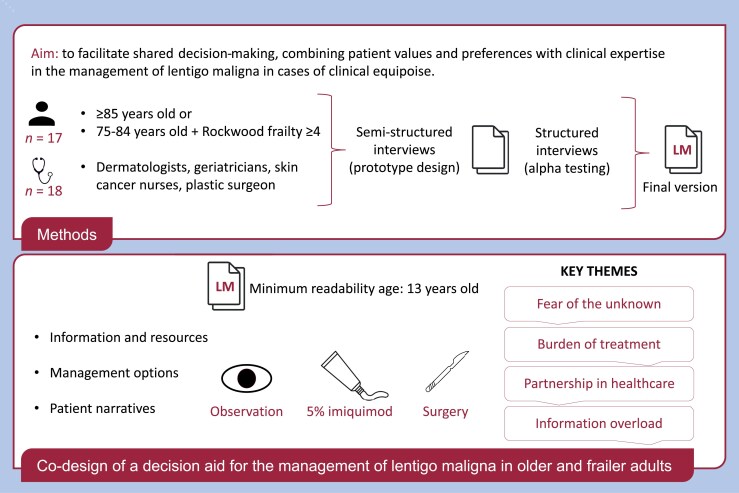

What is already known about this topic?

- Management options for lentigo maligna (LM) include surgery, imiquimod and observation.
- There is clinical equipoise on the best management of LM in older or frail adults.
- Patient decision aids are evidence-based, values-centred tools that empower patients to participate in treatment decisions and reduce decisional conflict.

What does this study add?

- This is the first decision aid for the management of LM in older or frailer patients; our study introduces a framework for co-designing patient decision aids.
- The four key themes identified can assist clinicians and empower older or frailer patients with LM in making shared management decisions.

Lentigo maligna (LM) usually presents later in life on ­sun-exposed skin and has extensive margins that often evade detection on dermoscopy.^1^ Treatment is aimed at preventing progression to lentigo maligna melanoma (LMM), and estimates for this vary from 10 to 50 years (mean 28), 3.5% per year (confidence interval 2.0–5.0%) or a 10-year cumulative risk of 0.7–1.1%.^2–4^ The natural progression of LM is challenging to study, as most lesions are excised upon diagnosis and studies use different methodologies, from self-reporting in patient questionnaires to nationally registered incidence data on LMM after LM.^2–4^ Although wide local excision with 5-mm margins is the standard of practice in the UK, Europe and the USA (North American guidelines consider extension up to 1 cm for LM, where possible), Guitera *et al.* showed, using reflectance confocal microscopy, that 59% of patients had subclinical disease beyond the recommended excision margins resulting in high incomplete excision rates, requiring complex surgery with cosmetic morbidity.^5–8^ Alternatives to surgery include observation or topical imiquimod, which achieves clearance rates of around 78%, largely depending on the application regiment.^9–11^ Nowadays, the trend to observe or treat topically may reflect the stabilizing crude incidence of LM in England between 2013 and 2019, in contrast to other melanomas *in situ*, which are increasing in incidence.^12^

The most appropriate management of LM in frail patients and those with multiple comorbidities or limited life expectancy remains uncertain.^13^ The risks of surgery are amplified in these patient groups, who have unique healthcare needs and are often excluded from shared ­decision-making.^14^ Given the slow estimated progression of LM and the burden of surgical treatment, a tailored approach is needed, combining clinical expertise with patient preferences. Most patients with melanoma prefer having an active role in treatment decisions.^15^ Patient ­decision aids (PDAs) are evidence-based tools that empower patients, foster partnerships with their healthcare professionals (HCPs) and enable patients to participate actively in treatment discussions.^16^ Co-design is a philosophy and method that refers to the application of user-centric research and service development approaches to solve a particular problem.^17^ It is dynamic, embracing partnership with community and focusing on systems change and improving human experience.^18^

The aim of this qualitative study was to co-design the first PDA for older or frail adults with LM, to facilitate shared decision-making when deciding on management. In 2021, the UK government emphasized the importance of prioritizing patient-centred research.^19^ Currently, there is no PDA available for older or frail adults with LM, the age group most commonly affected by LM, and for whom there is clinical equipoise on treatment.

## Patients and methods

This was a qualitative study using participant interviews to co-design a PDA following the International Patient Decision Aids Standards (IPDAS) Collaboration framework and in line with the Standards for Reporting Qualitative Research (SRQR) checklist (Appendix S1; see Supporting Information).^20,21^ This study focused on the first three steps of the IPDAS framework: (i) scoping and design; (ii) prototype formation; and (iii) ‘alpha’ testing (for accuracy and usability).

### Scoping and design

The PDA was targeted at patients aged at least 85 years or those aged between 75 and 84 years with a Rockwood Clinical Frailty Score (CFS) of at least 4, in light of the recognized need to provide bespoke treatment choices for those with potentially limited life expectancy.^22^ Data from the Office for National Statistics (UK) show that the average life expectancy at age 85 years is 5.7 years for men and 6.8 years for women, with a probability of dying in the next year of 10.0% and 7.5%, respectively.^23^ The CFS has been widely validated, is a predictor of mortality and its use in many ­preoperative assessment pathways is evidence based.^24,25^ A CFS ≥4 is defined as ‘vulnerable’ or ‘very mildly frail’.^26^ To minimize inaccurate patient recall, only those with a diagnosis of LM in the last 5 years were included.

The eligibility criteria were therefore (i) patients with a clinical or histological diagnosis of LM in the past 5 years and who were 85 years of age or older at the time of diagnosis, or those aged 75–84 years with a CFS ≥4 at the time of diagnosis; (ii) consultant dermatologists, geriatricians, plastic surgeons and specialist skin cancer nurses within the Norfolk and Norwich University Hospital NHS Foundation Trust; and (iii) able to read and speak English.

Eligible patients were identified retrospectively, by confidentially accessing their electronic healthcare records in chronological order and through proceedings of skin cancer multidisciplinary team discussions. Patients with other types of melanoma *in situ* or those with LM diagnosed over 5 years ago were excluded. Eligible patients were called to discuss the study with D.K., and a patient information leaflet was subsequently mailed to those expressing an interest (Appendix S2; see Supporting Information). Two weeks later, D.K. contacted the patients to obtain written consent (Appendix S3; see Supporting Information). HCPs were contacted through confidential email communications or grand round presentations (Appendixes S4, S5; see Supporting Information).

### Prototype formation

To design the prototype, two dermatology residents (D.K. and K.M.) held semi-structured, individual, nonrecorded, interviews with patients and HCPs. Patients were asked about the individual, social and cultural factors behind their decision for treatment, their information needs and narratives, and their overall concerns, experiences and preferences with regard to management of LM. HCPs were asked about structural and content elements of the PDA, including material for inclusion, nomenclature, definitions and treatment options. At the end of each interview, the interviewers presented potential material for inclusion in the PDA and asked participants for their feedback. All interviews were coded and analysed by D.K. and K.M. concurrently with data collection, using NVivo 14 (https://lumivero.com/products/nvivo/), for thematic analysis.^27,28^ More feedback was obtained through grand round presentations and dermatology departmental research meetings. Synthesized member-checking was used to enhance trustworthiness, with participants confirming the credibility of themes and subthemes, and by sending the final version of the PDA to all participants for feedback.^29^

### Alpha testing

Structured, nonrecorded, individual interviews with patients and HCPs were conducted by D.K. and K.M. to gather feedback on the usability and accuracy of the prototype, as in Junn *et al.*^30^ Participants were recruited as above, including four patients who met the eligibility criteria but did not have a prior diagnosis of LM, to simulate the level of understanding of a patient at the time of a new diagnosis of LM. The PDA was amended after every interview in an iterative process, until no further amendments were raised during the interviews and saturation was achieved.

## Results

**Figure 1:**
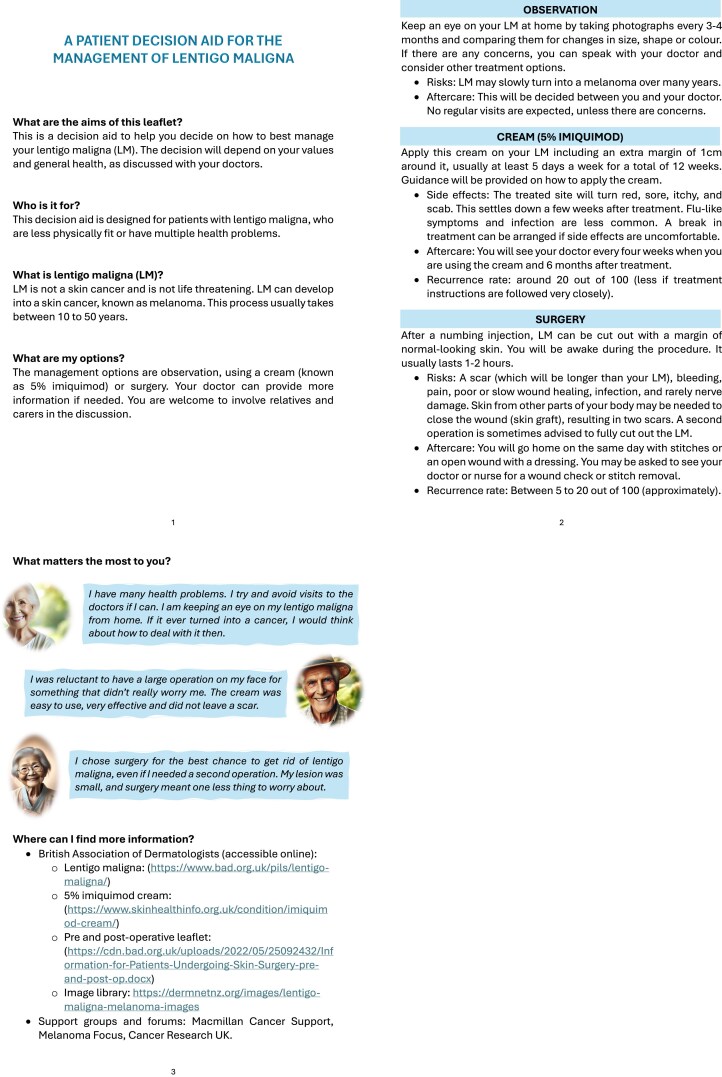
The patient decision aid for older or frailer patients with lentigo maligna.

A total of 22 patients and 25 clinicians were contacted, with 13 patients and 18 HCPs agreeing to participate to co-design the prototype (Table 1). The median interview duration was 27 min (range 19–39). Patient characteristics are described in Table 2. The median age was 83 years (range 77–88) and median CFS 4 (range 2–6). Four key themes emerged from the analysis and are discussed below, with subthemes and topics shown in Table 3. A total of 20 prototype iterations were required to achieve saturation and develop the final PDA (Figure 1). The PDA is written at a maximum reading level of 13 years old [assessed using the Simple Measure of Gobbledygook (SMOG) index, Flesch Kinaid formula and Automated Readability index]. The Standards for UNiversal reporting of patient Decision Aid Evaluation (SUNDAE) guidelines for evaluating PDA studies are available in Appendix S6 (see Supporting Information). There were no unanticipated consequences of using the PDA.

**Table 1:** Patients and healthcare providers, by specialty, participating in the different stages of co-designing the patient decision aid

	Patients (*n*)	Geriatricians (*n*)	Dermatologists (*n*)	Plastic surgeons (*n*)	Specialist nurses (*n*)
Protype formation	13	5	9	1	3
Alpha testing	7	3	7	0	3

All nurses who participated were specialists in skin cancer; all other healthcare providers were consultants or equivalent.

**Table 2:** Characteristics of the patients involved in the prototype design

Patient ID	Sex	Age (years)	Ethnicity	CFS	LM site	Management
P1	Female	87	White	2	Upper arm	Surgery + WLE
P2	Male	85	White	2	Cheek	Surgery + WLE
P3	Female	88	White	4	Forearm	Surgery + WLE
P4	Female	81	White	6	Forearm	^1^Surgery + ^2^imiquimod for recurrence
P5	Male	88	White	6	Upper arm	Surgery
P6	Female	86	White	2	Forehead	Surgery + WLE
P7	Male	77	White	4	Cheek	Imiquimod
P8	Female	78	White	4	Forehead	^1^Surgery + WLE ^2^imiquimod for recurrence
P9	Male	87	White	3	Cheek	Imiquimod
P10	Male	81	White	4	Cheek	Imiquimod
P11	Male	79	White	4	Nose	Imiquimod
P12	Male	88	White	3	Cheek	Observation
P13	Male	77	White	4	Nose	Imiquimod

All surgeries were standard excisions with a target 5-mm margins. Superscripts (^1,2^) show the order of management if more than one treatment was needed. CFS, Rockwood Clinical Frailty Score; LM, lentigo maligna; WLE, wide local excision.

**Table 3:** Overview of themes, subthemes and topics that emerging from the analysis of interviews of patients and healthcare providers

Themes	Subthemes	Topics
1. Fear of the unknown	Definition	What is LM? Is it cancer?
	Risk of progression to LMM	How common is LM?
	Prognosis with treatment	Is treatment effective?
		Necessity to treat asymptomatic lesions
2. Burden of treatment	Risks, side effects and delays in treatment	Convenience of treatment
	Likelihood of recurrence	Cosmetic outcomes
	Frequency of follow-up	List of recommended options
	Impact on QoL	Risks with and without treatment
		Shortages and barriers to treatment
3. Partnership in healthcare	Empowering patients and involving family and carers	Avoid medical jargon and stigmatizing terminology
	Burden of responsibility	Include visual aids
		Personalize treatment
		Support groups
		Other patients’ stories
		Outsourcing decisions to the doctor and the balance of anxiety of choice versus autonomy
4. Information overload	Less is more	Simple and concise
	Accessibility	Limited capacity to register information
	Optional additional resources	Print and online formats
		Large font size for those with visual impairment
		Multiple information leaflets for comorbidities
		Prevent misinformation and reduce information anxiety

LM, lentigo maligna; LMM, lentigo maligna melanoma; QoL, quality of life.

### Theme 1: fear of the unknown

‘Is it cancer?’ (Patients 4, 5, 9, 12).

‘Is treatment going to be successful?’ (Patient 13).

This was a common question asked in the interviews. Some patients did not understand what the diagnosis of LM meant. Others reported not being worried about their lesion, due to ‘bigger health problems’. The terms used by patients to describe their understanding of the condition varied significantly: ‘age spot’, ‘pre-cancer’, ‘slow growing cancer’, ‘early stage of cancer’, ‘dormant form of cancer’, ‘skin cancer’ and ‘melanoma’. Most patients opted for treatment due to the fear of cancer, while others had treatment only based on their clinician’s recommendation. There was unanimous agreement to define LM in simple terms in the PDA to aid understanding of the diagnosis for future patients. Through iterations, the PDA explicitly states that ‘LM is not a skin cancer…’ followed by information on progression, to help patients quantify the known risks. This information is presented as absolute numbers (and not percentages or pictograms), as per patient preference.

### Theme 2: burden of treatment

‘If I could turn back time, I would have left it alone’ (Patient 4).

‘If it [the LM] were in a different position, I would have thought twice before surgery’ (Patient 1).

Some patients discontinued imiquimod due to severe pain, while one patient did not expect a wide local excision and subsequently a ‘long scar’. All but one patient reported the need to clearly outline the time needed for each treatment and recovery, any ‘side effects’, and follow-up visits or further procedures that may be necessary. One patient did not think that the burden of treatment was important to capture in the PDA due to the risks of information overload. Most patients and HCPs agreed that transparency with regard to the risks of each management option are essential in facilitating informed and shared ­decision-making. This is reflected by including the expected, most common and most burdensome risks in the PDA, along with any further time commitments necessary, such as follow-up visits.

### Theme 3: partnership in healthcare

‘The doctor said that I ought to have surgery, so I went with it’ (Patient 3).

Management for most patients was decided by their HCP. While some were not offered a consultation to voice their preferences on management, others chose to leave the decision in the hands of their doctors, and only two patients reported involving family or friends in their decision. Most would have preferred to participate in decision-making but found it difficult to comprehend and weigh the different management options. One participant suggested using hypothetical narratives in the PDA to help patients associate with fictional patients, empowering them in the process of decision making. This addition was well received by HCPs and other patients. Patients preferred a written copy of the PDA, while most clinicians opted for online access and therefore the PDA was produced in both formats.

### Theme 4: information overload

‘I got a leaflet with all the information, but I never read it’ (Patient 10).

‘Keep it simple’ (Patients 2, 5, 10, 11, 12).

The uncertainty of diagnosis that heralded the fear of cancer was reinforced by ‘too much information’ provided to patients. Most patients received verbal and written (in the form of patient information leaflets) information, along with online resources on LM. This creates a paradox where excessive information may reduce motivation in those older or with multiple comorbidities to carefully read the content, thereby resulting in deferring the decision of management to their HCP.

Some clinicians and patients advised against using references in the PDA. However, other patients expressed a preference to compile a concise reference list for those wishing to know more, and their families. Therefore, five key references were included at the end of the PDA, to direct patients to more information on treatment options and postoperative recovery, support groups and an image library on LM.

## Discussion

This study used co-design to develop and test a PDA for older or frail patients with LM, following the IPDAS Collaboration framework. Integrating patients throughout the development cycle improves the content validity and acceptability of the PDA, while reconciling epistemic gaps between clinical and experiential knowledge, thereby strengthening the tool’s potential for implementation and impact in shared ­decision-making.^31,32^ PDAs hold an important role in ­dermatology, respect the ethical principle of autonomy and studies in patients with melanoma show that more than 80% of patients wish to actively participate in treatment decisions.^15,33^ Semi-structured interviews raised key points that were identified during thematic analysis and refined in the PDA during the iterative alpha-testing process, until saturation was achieved. Patient feedback ensured the design focused on patient values, understanding and addressing the barriers to partnership in healthcare. The diversity of clinical specialists allowed for different perspectives of experts involved in the care of patients with LM and maintained the accuracy of the contents of the PDA. The format used in this study may be helpful in co-designing PDAs for other conditions in the future.

The fear of cancer and uncertainty of diagnosis was a unanimous theme and hurdle to patient participation in ­decision-making. This is addressed using jargon-free definitions within the first page of the PDA and simple explanations of the known risks and time to progression. Patients are encouraged to involve friends and family in the decision-­making process, time is allowed for patients to reflect on their options and ask questions before reaching a decision, and the option to switch to a different choice of management is offered if patients’ needs or priorities change over time. A flowchart summarizing the available management options was originally included and well received by HCPs but was ultimately omitted as several patients reported difficulty in understanding it (Appendix S7; see Supporting Information). This disagreement highlights the importance of co-design. As Robert Pirsig argued, technical expertise alone may lead to the development of a tool, but its uptake, engagement with the tool and perceived usefulness all require the in-built values of its intended target audience.^34^ The second page addresses the theme of ‘burden of treatment’, by comparing the risks and follow-up of three different management options. Early iterations displayed this information in table format, which was gradually replaced by concise sections of text, following feedback from patients and HCPs on simplicity and design, respectively. The third page comprises hypothetical patient narratives alongside images generated by ChatGPT 3.5, followed by a brief list of optional references, including an image library of LM and more details on management. The importance of values clarification in PDAs is well known and patients in this study reported that the hypothetical narratives would reduce decisional conflict and possibly consultation time in clinic.^35^ The large font, paragraph spacing, document length and visual cues make the PDA more accessible and limit information overload, which was another theme patients identified as a barrier to shared decision-making.

Limitations include that the study was conducted only in the East of England, involving a small number of patients of the same ethnicity, and HCPs from three hospitals in one region. The images in the PDA do not include skin of colour, where LM is a rare finding. While educational background can affect PDA outcomes and was not recorded in this study, the PDA is written at a maximum reading level of 13 years old.^36^ The tool is targeted specifically at older or frail adults and is not intended for use in other patient groups, or where there is no clinical equipoise in treatment. Only a single patient who opted for observation was included in the study and only one plastic surgeon, with an interest in melanoma. The PDA only includes pragmatic and evidence-based treatments available for LM in this patient group. Therefore, (slow) Mohs surgery and alternative options (such as radiotherapy, cryotherapy and lasers) are not discussed. Albeit available in both paper and electronic formats, the PDA lacks videos or other interactive tools at present.

This is the first study to present a PDA for the management of LM in older or frail adults. The co-design process ensures relevance to patient values and perspectives, as well as clinical accuracy. The iterative development improves the comprehensibility and usability of the tool. Beta-testing, to follow, will determine the performance of the PDA in dermatology services with regard to patient participation in treatment decisions, satisfaction, outcomes and consultation times.

## Supplementary Material

vzaf118_Supplementary_Data

## Data Availability

The data underlying this article will be shared on reasonable request to the corresponding author, respecting patient confidentiality.
