# Supplementary material for: Co-design of a decision aid for the management of lentigo maligna in older and frailer adults
Source: Skin Health Dis. 2026 Mar 20;6(3):265–72. doi: 10.1093/skinhd/vzaf118 (PMC13220014; doi:10.1093/skinhd/vzaf118)

Appendix S1: Standards for Reporting Qualitative Research (SRQR) Equator Checklist

|  |  | **Page** |
| --- | --- | --- |
| **Title and abstract** | |  |
|  | **Title** - Concise description of the nature and topic of the study Identifying the study as qualitative or indicating the approach (e.g., ethnography, grounded theory) or data collection methods (e.g., interview, focus group) is recommended | 4 |
|  | **Abstract** - Summary of key elements of the study using the abstract format of the intended publication; typically includes background, purpose, methods, results, and conclusions | 2 |
|  |  |  |
| **Introduction** | |  |
|  | **Problem formulation** - Description and significance of the problem/phenomenon studied; review of relevant theory and empirical work; problem statement | 4 |
|  | **Purpose or research questio**n - Purpose of the study and specific objectives or questions | 4 |
|  |  |  |
| **Methods** | |  |
|  | **Qualitative approach and research paradigm** - Qualitative approach (e.g., ethnography, grounded theory, case study, phenomenology, narrative research) and guiding theory if appropriate; identifying the research paradigm (e.g., postpositivist, constructivist/ interpretivist) is also recommended; rationale** | 4-5 |
|  | **Researcher characteristics and reflexivity** - Researchers’ characteristics that may influence the research, including personal attributes, qualifications/experience, relationship with participants, assumptions, and/or presuppositions; potential or actual interaction between researchers’ characteristics and the research questions, approach, methods, results, and/or transferability | 5 |
|  | **Context** - Setting/site and salient contextual factors; rationale** | 5 |
|  | **Sampling strategy** - How and why research participants, documents, or events were selected; criteria for deciding when no further sampling was necessary (e.g., sampling saturation); rationale** | 4-5 |
|  | **Ethical issues pertaining to human subjects** - Documentation of approval by an appropriate ethics review board and participant consent, or explanation for lack thereof; other confidentiality and data security issues | 1, 4 |
|  | **Data collection methods** - Types of data collected; details of data collection procedures including (as appropriate) start and stop dates of data collection and analysis, iterative process, triangulation of sources/methods, and modification of procedures in response to evolving study findings; rationale** | 4-5 |
|  | **Data collection instruments and technologies** - Description of instruments (e.g., interview guides, questionnaires) and devices (e.g., audio recorders) used for data collection; if/how the instrument(s) changed over the course of the study | 4-5 |
|  | **Units of study** - Number and relevant characteristics of participants, documents, or events included in the study; level of participation (could be reported in results) | 5,11 |
|  | **Data processing** - Methods for processing data prior to and during analysis, including transcription, data entry, data management and security, verification of data integrity, data coding, and anonymization/de-identification of excerpts | 5 |
|  | **Data analysis** - Process by which inferences, themes, etc., were identified and developed, including the researchers involved in data analysis; usually references a specific paradigm or approach; rationale** | 5 |
|  | **Techniques to enhance trustworthiness** - Techniques to enhance trustworthiness and credibility of data analysis (e.g., member checking, audit trail, triangulation); rationale** | 5 |
|  |  |  |
| **Results/findings** | |  |
|  | **Synthesis and interpretation** - Main findings (e.g., interpretations, inferences, and themes); might include development of a theory or model, or integration with prior research or theory | 5, 7-8 |
|  | **Links to empirical data** - Evidence (e.g., quotes, field notes, text excerpts, photographs) to substantiate analytic findings | 6-7 |
|  |  |  |
| **Discussion** | |  |
|  | **Integration with prior work, implications, transferability, and contribution(s) to the field -** Short summary of main findings; explanation of how findings and conclusions connect to, support, elaborate on, or challenge conclusions of earlier scholarship; discussion of scope of application/generalizability; identification of unique contribution(s) to scholarship in a discipline or field | 7-8 |
|  | **Limitations** - Trustworthiness and limitations of findings | 8 |
|  |  |  |
| **Other** | |  |
|  | **Conflicts of interest** - Potential sources of influence or perceived influence on study conduct and conclusions; how these were managed | 1 |
|  | **Funding** - Sources of funding and other support; role of funders in data collection, interpretation, and reporting | 1 |

Appendix S2: The patient information leaflet.

**PARTICIPANT INFORMATION SHEET**

**Development of a decision aid for managing lentigo maligna in older patients or patients with limited life expectancy.**

You are being invited to take part in a research study. Before deciding it is important to understand why the research is being done and what this study involves. Please take time to read the following information carefully. Please ask us if you have any questions or if you would like more information.

What is the purpose of the study?

To develop a decision aid to help older patients and those with limited life expectancy decide on the management of their lentigo maligna. Patient decision aids are tools scientifically proven to help patients make specific healthcare decisions and participate in shared decision making.

What is lentigo maligna?

Lentigo maligna is a slow growing skin cancer that appears later in life, usually on the head or neck. It is sometimes referred to as “melanoma *in* situ”, because it affects only the surface of the skin. Over several years however, lentigo maligna may slowly move deeper into the skin and become an invasive skin cancer (known as “malignant melanoma”). The risk of this is poorly understood and may take 10 to 40 years (but also more or less) depending on several factors, such as age at diagnosis. A diagnosis of lentigo maligna does not shorten life expectancy.

Surgery can best prevent this risk more than other treatment options available. However, is surgery the best treatment for older or more frail patients? The benefit of each operation needs to be weighed against the risks of surgery (mainly bleeding, infection, poor healing and incomplete removal of the lesion) and the slow growth of lentigo maligna.

What’s involved?

Twenty patients and 20 healthcare professionals will be interviewed to create a decision aid (available in print and online formats) that reflects patient values, is accurate and usable. The research team will revise the decision aid after each interview, to reflect patient narratives and clinician experiences. The aim is to create a user-friendly and valid tool to support older adults and patients with limited life expectancy in making decisions on the management of their lentigo maligna.

To confirm your participation, you will be asked to sign a consent form only after you have had enough time to make an informed decision. You will then be contacted by a member of the research team to arrange one interview. The interview will be with a clinician-member of the research team and will not be recorded. It will take place either as a phone call or video call, in private. If you prefer, your carer can help you with the interview (examples include setting up the video call or phone call, or repeating questions that may not sound clear over the phone). We expect the interview to last between 20 and 40 minutes but this may vary.

Participation is voluntary. As a participant you can withdraw at any time, without any adverse consequences. You can withdraw by contacting Dr Karponis at any point ([Dimitrios.karponis@nnuh.nhs.uk](mailto:Dimitrios.karponis@nnuh.nhs.uk)). Any data collected up until you withdraw will be held securely and confidentially as per the Norfolk and Norwich University Hospital NHS Trust policy, until the permanent deletion date in 5 years.

Who can take part?

To participate in this study you will need to be able to read and speak in English, be 85 years or older, or 75 years or older with a Rockwood Frailty Scale of at least 4 (this is a score doctors often use to assesses your general fitness). If you have lentigo maligna, your diagnosis will need to have been made in the last 5 years to be eligible for the study.

The possible benefits of taking part.

By participating, you will help in the creation of a tool to empower patients decide on the management of their lentigo maligna and learn more about lentigo maligna.

The possible risks of taking part.

There are no foreseeable harms.

How participants’ data are processed in line with GDPR.

In this research study we will use information from you and your medical records. We will only use information that we need for the research study. We will let very few people know your name or contact details, and only if they really need it for this study.

This information will include your NHS number, name, contact details, age, fitness status (if available), and skin conditions in your medical records.  People will use this information to do the research or to check your records to make sure that the research is being done properly.

Everyone involved in this study will keep your data safe and secure. We will also follow all privacy rules.

At the end of the study we will save some of the data in case we need to check it. We will make sure no-one can work out who you are from the reports we write.

We will keep all information about you safe and secure.

Once we have finished the study, we will keep some of the data so we can check the results. We will write our reports in a way that no-one can work out that you took part in the study. You can stop being part of the study at any time, without giving a reason, but we will keep information about you that we already have. We need to manage your records in specific ways for the research to be reliable. This means that we won’t be able to let you see or change the data we hold about you.

You can find out more about how we use your information:

- at [www.hra.nhs.uk/information-about-patients/](https://www.hra.nhs.uk/information-about-patients/)
- by asking one of the research team, or
- by ringing us on 01603 647882, or
- by contacting the data protection officer at [info.gov@nnuh.nhs.uk](mailto:info.gov@nnuh.nhs.uk)

Further supporting information.

The study is organised by the Norfolk and Norwich University Hospital. Any information collected during the interviews will be locked in a filing cabinet at the Department of Dermatology, Norfolk and Norwich University Hospital, accessible only to the research team.

At the end of the study, we aim to publish our results, without revealing any information that can be used to identify you in any way. Your data will not be used for any type of future research without first obtaining consent from you.

The study has been reviewed by the Norfolk and Norwich University Hospital Research and Development office and the Healthcare Research Authority.

For any further information about the study, or if you wish to submit a complaint, please contact Dr Karponis ([Dimitrios.karponis@nnuh.nhs.uk](mailto:Dimitrios.karponis@nnuh.nhs.uk)) or the Norfolk and Norwich University Hospital Patient Advice and Liaison Service (PALS) on 01603 289036 (or via email: [palsandcomplaints@nnuh.nhs.uk](mailto:palsandcomplaints@nnuh.nhs.uk)).

Appendix S3: A copy of the patient consent form.

IRAS ID: 347670

Centre Number:

Study Number:

Participant Identification Number for this trial:

**CONSENT FORM**

Title of Project: Development of a patient decision aid for the management of lentigo maligna in frail adults.

Name of Researcher: Dr Dimitrios Karponis

Please initial box

1. I confirm that I have read the information sheet dated.................... (version...........) for the
   above study. I have had the opportunity to consider the information, ask questions and have
   had these answered satisfactorily.
2. I understand that my participation is voluntary and that I am free to withdraw at any time
   without giving any reason, without my medical care or legal rights being affected.
3. I understand that relevant sections of my medical notes and data collected during
   the study may be looked at by individuals from the Norfolk and Norwich University Hospital,
   where it is relevant to my taking part in this research. I give permission for
   these individuals to have access to my records.
4. I understand that the information collected about me will be used to support
   other research in the future and may be shared anonymously with other researchers.
5. I understand that the information held and maintained by the Norfolk and Norwich
   University Hospitals NHS Foundation Trust may be used to help contact me
   or provide information about my health status.
6. I agree to take part in the above study.

Name of Participant Date Signature

Name of Person Date Signature

seeking consent

Appendix S4: The healthcare practitioner information leaflet.

**PARTICIPANT INFORMATION SHEET**

**Development of a decision aid for managing lentigo maligna in older patients or patients with limited life expectancy.**

You are being invited to take part in a research study. Before deciding it is important to understand why the research is being done and what this study involves. Please take time to read the following information carefully. Please ask us if you have any questions or if you would like more information.

What is the purpose of the study?

To develop a decision aid to help older patients and those with limited life expectancy decide on the management of their lentigo maligna. Patient decision aids are tools scientifically proven to help patients make specific healthcare decisions and participate in shared decision making.

What is lentigo maligna?

Lentigo maligna is a slow growing skin cancer that appears later in life, usually on the head or neck. It is sometimes referred to as “melanoma in situ”, because it affects only the surface of the skin. Over several years however, lentigo maligna may slowly move deeper into the skin and become an invasive skin cancer (known as “malignant melanoma”). The risk of this is poorly understood and may take 10 to 40 years (but also more or less) depending on several factors, such as age at diagnosis. A diagnosis of lentigo maligna does not shorten life expectancy.

Surgery can best prevent this risk more than other treatment options available. However, is surgery the best treatment for older or more frail patients? The benefit of each operation needs to be weighed against the risks of surgery (mainly bleeding, infection, poor healing and incomplete removal of the lesion) and the slow growth of lentigo maligna.

What’s involved?

Twenty patients and 20 healthcare professionals will be interviewed to create a decision aid (available in print and online formats) that reflects patient values, is accurate and usable. The research team will revise the decision aid after each interview, to reflect patient narratives and clinician experiences. The aim is to create a user-friendly and valid tool to support older adults and patients with limited life expectancy in making decisions on the management of their lentigo maligna.

To confirm your participation, you will be asked to sign a consent form only after you have had enough time to make an informed decision. You will then be contacted by a member of the research team to arrange one interview. The interview will be with a clinician-member of the research team and will not be recorded. It will take place either as a phone call or video call, in private. We expect the interview to last between 20 and 40 minutes but this may vary.

Participation is voluntary. As a participant you can withdraw at any time, without any adverse consequences.

Who can take part?

To participate in this study you will need to belong in one of the following categories, in the Norfolk and Norwich University Hospitals NHS Foundation Trust: consultant dermatologist, consultant plastic or Mohs surgeon, consultant geriatrician or skin cancer specialist nurse.

The possible benefits of taking part.

By participating, you will help in the creation of a tool to empower patients decide on the management of their lentigo maligna and learn more about lentigo maligna.

The possible risks of taking part.

There are no foreseeable harms.

How participants’ data are processed in line with GDPR.

In this research study we will use information from you. We will only use information that we need for the research study. We will let very few people know your name or contact details, and only if they really need it for this study.

This information will include your name and contact details.  People will use this information to make sure that the research is being done properly.

Everyone involved in this study will keep your data safe and secure. We will also follow all privacy rules.

At the end of the study we will save some of the data in case we need to check it. We will make sure no-one can work out who you are from the reports we write.

We will keep all information about you safe and secure.

Once we have finished the study, we will keep some of the data so we can check the results. We will write our reports in a way that no-one can work out that you took part in the study. You can stop being part of the study at any time, without giving a reason, but we will keep information about you that we already have. We need to manage your records in specific ways for the research to be reliable. This means that we won’t be able to let you see or change the data we hold about you.

You can find out more about how we use your information:

- at [www.hra.nhs.uk/information-about-patients/](https://www.hra.nhs.uk/information-about-patients/)
- by asking one of the research team, or
- by ringing us on 01603 647882.

Further supporting information.

The study is organised by the Norfolk and Norwich University Hospital. Any information collected during the interviews will be locked in a filing cabinet at the Department of Dermatology, Norfolk and Norwich University Hospital, accessible only to the research team.

At the end of the study, we aim to publish our results, without revealing any information that can be used to identify you in any way.

The study has been reviewed by the Norfolk and Norwich University Hospital Research and Development office and the Healthcare Research Authority.

For any further information, please contact Dr Dimitrios Karponis via email ([Dimitrios.karponis@nnuh.nhs.uk](mailto:Dimitrios.karponis@nnuh.nhs.uk)).

Appendix S5: A copy of the healthcare practitioner consent form.

IRAS ID: 347670

Centre Number:

Study Number:

Participant Identification Number for this trial:

**CONSENT FORM**

Title of Project: Development of a patient decision aid for the management of lentigo maligna in frail adults.

Name of Researcher: Dr Dimitrios Karponis

Please initial box

1. I confirm that I have read the information sheet dated.................... (version...........) for the
   above study. I have had the opportunity to consider the information, ask questions and have
   had these answered satisfactorily.
2. I understand that my participation is voluntary and that I am free to withdraw at any time
   without giving any reason, without my medical care or legal rights being affected.
3. I understand that the information collected about me will be used to support
   other research in the future and may be shared anonymously with other researchers.
4. I agree to take part in the above study.

Name of Participant Date Signature

Name of Person Date Signature

seeking consent

Appendix S6: The SUNDAE checklist.


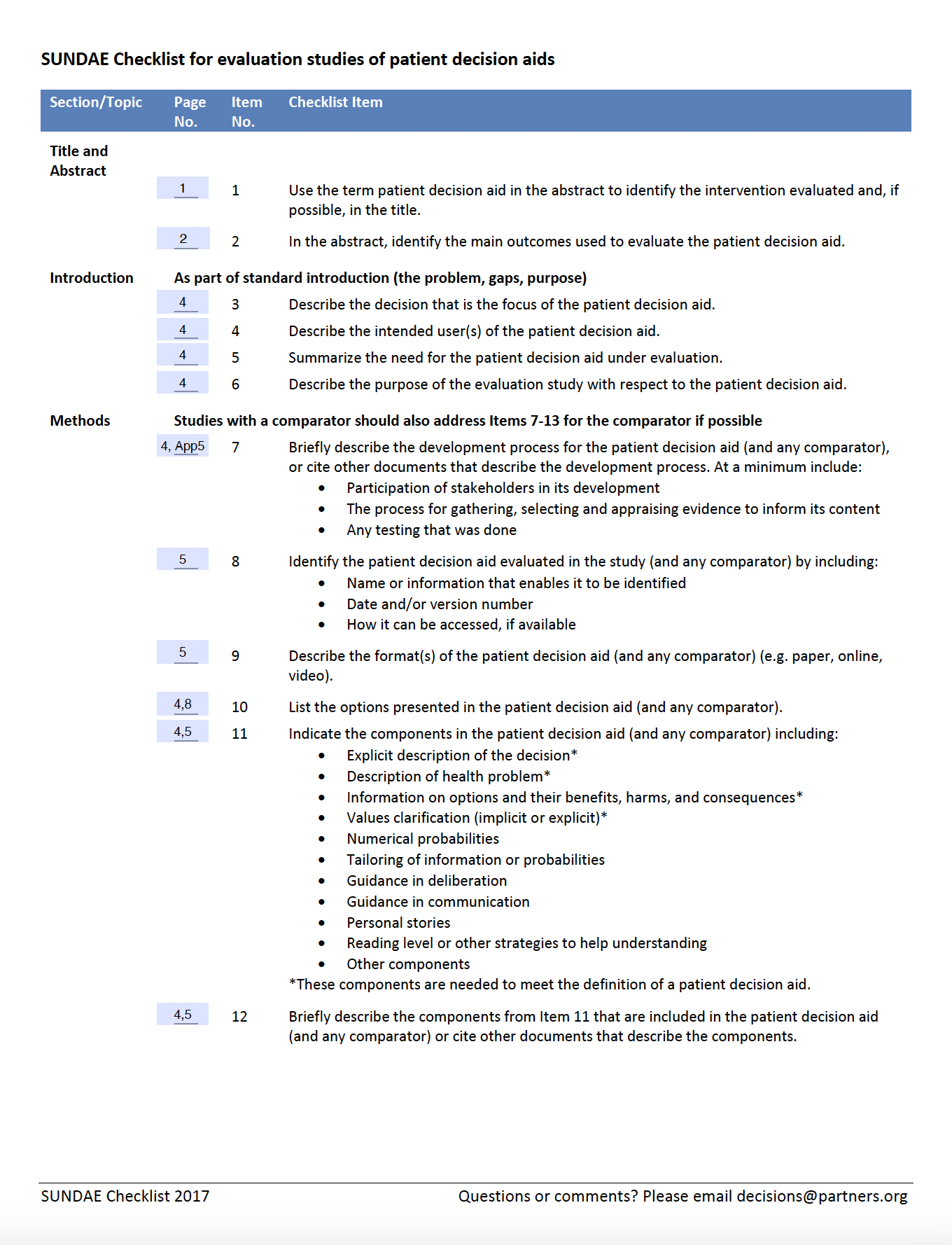


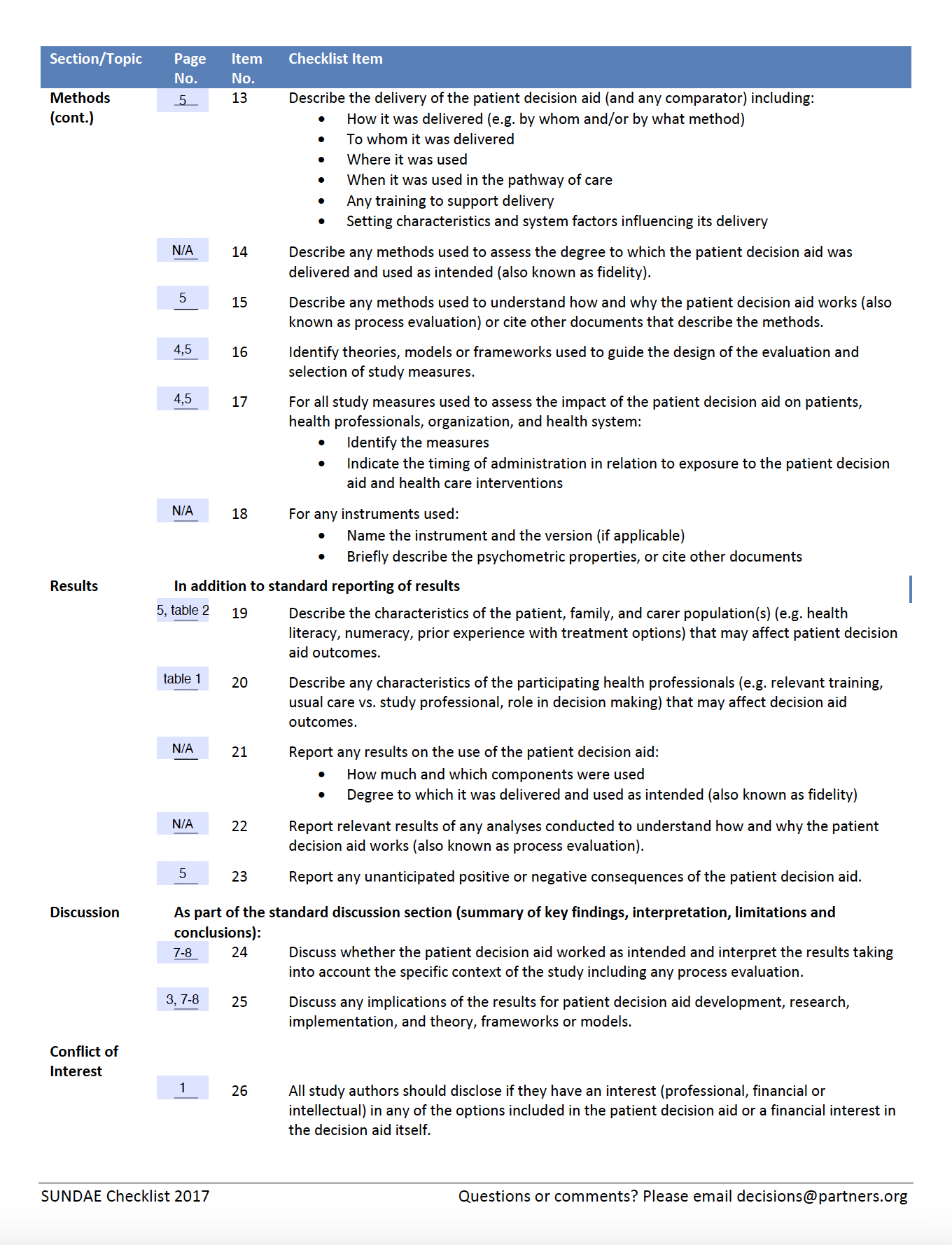


Appendix S7: A flowchart summarising the management options for lentigo maligna.


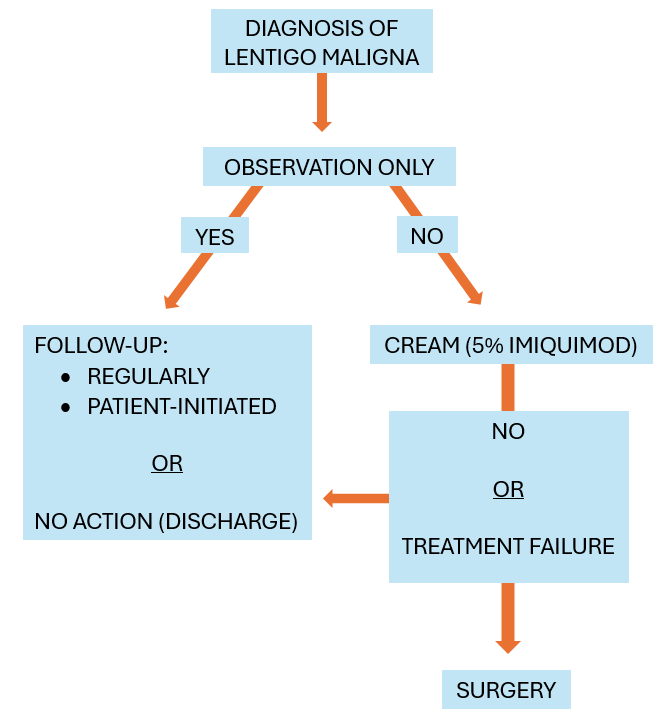

Supplement: vzaf118_Supplementary_Data [file vzaf118_supplementary_data.docx]
